# Leaving the Norwegian opioid maintenance treatment program - patient experiences

**DOI:** 10.1186/s12913-024-11859-3

**Published:** 2024-11-25

**Authors:** Sverre Nesvåg, Per Bergqvist, Ingrid Elin Dahlberg, James R. McKay

**Affiliations:** 1 Centre for Alcohol & Drug Research, Stavanger University Hospital, Stavanger, Norway; 2 PENN-TRI Centre on the Continuum of Care in the Addictions, University of Pennsylvania, Philadelphia, USA; 3 Philadelphia Veterans Affairs Medical Center, Philadelphia, USA

**Keywords:** Opioid maintenance treatment, Leaving OMT, Norway, Patient experiences

## Abstract

**Background:**

Opioid maintenance treatment (OMT) saves lives and makes it possible to start a process of health and social rehabilitation. Previous research shows that those who leave OMT after years of a drug free life and a reasonable level of health and social rehabilitation can have a good chance of living a stable drug free life after leaving the treatment. The aim of this study was to gain more knowledge about how patients who were in the process of leaving, or who had left OMT, experienced the leaving process.

**Methods:**

The study was based on a thematic analysis of transcripts from individual in-depth interviews with 24 patients who had experience with leaving the Norwegian OMT program. Results: The participants in this study had a strong motivation to endure a demanding substitution medication tapering process and leave the OMT program. The tapering benefited strongly from adaptive routines based on ongoing dialog between patients and service providers throughout the process, based on individualized goals and patient experiences each step on the way. The participants had varied experiences regarding the quality and relevance of the support and meeting the need for further treatment and services.

**Conclusions:**

Given a high level of rehabilitation and good support, this study shows that some OMT program patients are in a good position to successfully leave the OMT program or continue the substitution treatment on a low dosage in a less OMT program-dominated life.

**Supplementary Information:**

The online version contains supplementary material available at 10.1186/s12913-024-11859-3.

## Background

When opioid maintenance treatment (OMT) was introduced in the 1960’s [11], it was met with great optimism and soon became and still is, the first choice of treatment for opioid addiction. This optimism has found support in consistently positive findings, demonstrating reduced illicit opioid use, criminality, mortality and morbidity among OMT patients [7, 9, 14, 33, 35]. On the other hand, many OMT patients are still facing continuous socioeconomic difficulties, health issues, and problems related to other drug and alcohol use [42]. Medication is often given in connection with control regimes which are established to secure a medically sound treatment and stop leaking of OMT drugs to the illegal market. Many patients find such control regimes difficult to follow, as well as degrading and hard to accept [15, 16, 28]. This burden of OMT treatment [25] has contributed to treatment retention problems in many countries [29].

OMT is a treatment without any time limit. The very name, “maintenance treatment”, connotes a treatment that seeks to stabilize the patient in a less risky life situation but not eliminate dependence on an opioid. The successful attainment of treatment goals depends on continuing to take the substitution medication. Ideally, this should mean long term if not lifelong treatment. But, due to the difficulties with retention, many countries have had to accept shorter treatment lengths. For example, the US National Institute on Drug Abuse recommends that OMT should be utilized for a minimum of 12 months, and retention after 18 months is an emerging quality treatment indicator [1].

In some countries, as in the Norwegian OMT program, the aim of the treatment is more ambitious, both when it comes to treatment length and treatment effects beyond mere maintenance. In Norway, maintenance medication is part of a treatment program containing a menu of possible psychosocial treatment methods and social services. The aim of the program is also reflected in its name: Legemiddel Assistert Rehabilitering (Medication Assisted Rehabilitation), abbreviated LAR. LAR has also become the name of the special services organized to deliver the program, either directly from the LAR or from ordinary health and social services.

The Norwegian OMT program has a very high coverage, compared to most other countries. For example, while substitution treatment is offered to less than 15% of individuals with an opioid use disorder in the US [38], coverage in Norway is estimated to 86% [13]. The number of patients in 2022 was 8300 and has been very stable the last ten years. Retention in treatment is also very high. In 2022, only 7% of the patients left the treatment program (including deaths) [26].

Most research on retention in OMT and on what happens to those who leave treatment has focused on the small numbers of patients who manage to stay drug free after leaving treatment [27]. This is particularly the case in countries where retention in treatment is low [41]. Different studies show that between 5% and 18% of those who leave OMT, for any reason, managed to stay stably drug-free [10, 18, 27]. However, the reason for leaving strongly influences the success rate. The Norwegian study [10] showed that of the 469 patients in OMT in one hospital over a 10 year period, 103 left treatment. Of these, only 19 (4% of all patients and 18% of those who left) left because they considered themselves “to be drug-free and had completed the treatment” ([10], p. 1148). All of these 19 patients stayed drug-free at least two years after treatment, while almost none of the other 84 who left treatment, managed to do so.

Based on this research, it appears that most patients who leave OMT, regardless of why they leave, are not able to stay drug free. In addition, a high risk of relapse to opioids leads to an increased likelihood of overdose, as well as further health and social problems. On the other hand, the research also shows that those who leave OMT after years of a stable recovery from opioid addiction and a reasonable level of health and social rehabilitation are much more likely to be able to live a stable drug free life after leaving the treatment.

Research on what interventions can provide the most effective support and help for patients who want to leave OMT is limited. However, research dating from the 1980s documented the importance of a planned, slow substitution medication tapering, professional advice, good access to health and social services, and peer support for patients who wish to discontinue OMT [32]. These findings have been confirmed in later studies [5, 43]. New studies, however, have added little new knowledge to the study from 1984, even though the conclusion of this article called for more research, i.e., “- we need further systematic analyses of the coping mechanisms of those who have “made it” out.” ([32], p. 550). This was also the conclusion of a recent Norwegian survey [40].

The aim of the present study was to better understand the experiences and perceptions of patients who believed that they could maintain recovery from opioid addiction after leaving OMT. These patients were either in a process of leaving or had actually left the Norwegian OMT program. The goals of the study were to assess:

- Patients’ perceptions of their life and health situations and their relation to the OMT program when they decided to try to leave the treatment.
- The ways in which patients coped with the challenges they faced in the tapering of the OMT medication and when trying to leave the OMT program.
- Patients’ perceptions of the help and support they received while leaving OMT as well as after they had left the program.
- And finally, how they experienced their health and life situation after having tried to leave, had re-entered or had actually left the Norwegian OMT program.

Increasing our knowledge on what patients themselves find important in a process of trying to leave OMT is vital in developing services that can support patients in such a process.

## Methods

As the aim of the study was to gain a deeper understanding of the patients’ experiences of leaving the Norwegian OMT program, a qualitative approach was chosen as best methodological design for the study. The study was based on a thematic analysis of transcripts from individual in depth interviews with 24 patients who had tried to leave or had left Norwegian OMT.

### Researchers

The project team responsible for this study consists of four members with different education and experiences. The project leader is a social science professor with many years of research experience in the alcohol and drug field. Two of the members of the team have been patients in the Norwegian OMT program. One left the program ten years ago and is now working as a skilled worker in a building construction company and as a peer experience consultant in a municipality social service. He was recruited and trained as an interviewer and co-researcher in this project. The other member with lived experience in OMT is still a patient in OMT but has experiences with both tapering and change in substitution medication. She has been a co-researcher in numerous projects and has completed both co-research training and relevant formal academic education. She took an active part as a co-researcher in all phases of the project. The last member of the research group is a psychology professor with many years of research experience in the alcohol and drug field, including research on OMT in the US. His role has been as a research advisor and co-author of this report. With this composite of competencies, the research team was able to conduct a real co-research project, involving both clinical researchers and individuals with lived experience as OMT patients [2, 39].

### Participants

The 24 participants in the study were recruited from all of the four major health service regions of Norway; eleven from the west, seven from the south-east, three from the central and three from the north. They were recruited to the study in different ways. As this study started as a health service research project in one hospital in the western region, the eleven participants from this region were recruited either via a consultant in the OMT program department in the hospital or via participants already recruited to the study. The study then developed into a health service research project also covering the three other health regions. The seven participants from the south-eastern region (by far the largest of the regions), the three participants from the central region, and the three participants from the northern region of Norway were all recruited via contact persons in the regional substance user organizations. The OMT program departments in these regions were also contacted and asked for help in recruiting participants but were not able to do so. None of the OMT program patients or former OMT program patients who were asked to participate declined to do so.

### The interviews

Two years prior to this study, one member of the research group conducted an unpublished pilot study, based on qualitative interviews with seven former or current Norwegian OMT patients, that focused on their experiences as OMT patients and on leaving/re-entering OMT. This study was important in the planning of the study reported in this paper, with regard to both the strategy for recruiting participants and the semi-structured interview guide (see Additional file).

The 24 interviews were conducted by the one of the members of the project group with lived experience of illegal opioid use and OMT treatment. Prior to the interviews he went through an internal education and training in how to conduct good qualitative interviews, with a special focus on how to avoid own experiences and prejudices affect how questions are asked and reacted to. The interviews lasted between 30 and 60 min and were all audio taped. The interviews were conducted with the help of an interview guide that ensured that all questions areas were covered in all interviews and facilitated further conversation to dig deeper into areas of special importance to each participant. The OMT patient lived experience of the interviewer made it easier for the participants to relate to him and to reveal emotions and experiences that are often difficult to elicit in single interview situations. The interviewer`s combination of patient experience and co-researcher competence also made it easier for him to understand what was said and ask for more precise information and deeper reflections.

### Analysis

The audiotapes from the interviews were all transcribed in full text and then analysed, following the steps for a thematic analysis recommended by Braun & Clark [6]: (a) familiarization with the data, (b) coding, (c) searching for themes, (d) reviewing the themes, (e) defining and naming the themes, and (f) writing up the report. QSR NVivo was used as a practical tool in the analysing process.

In the first five steps of this process, the first author and the two members of the project group with lived experience as OMT patients took an equal, active part. In the writing process the first author had the main responsibility for writing the first draft of the manuscript and the other members making contributions in the process and to editing of subsequent drafts, in accordance with the Vancouver criteria for co-authorship.

The analysis used an explorative and reflexive approach [3] where the experiences of the participants were interpreted and reinterpreted in the view of how we in the project group understand the dynamic process of clinical and personal recovery from severe opioid addiction. This process benefited from the various types of expertise within the project group. The patient expertise was important in determining that our interpretations of the data and derivations of themes were similar to the experiences of the participants. The scientific expertise was also important in following sound principles of qualitative research design and analysis and in the discussion of the external validity of the study [24].

### Ethics

In accordance with the Norwegian regulation of health service research projects, the study was evaluated and approved by the Data protection officer and the Research department of the hospital responsible for the study, Stavanger University Hospital (ID 2018-5). Written informed consent was obtained from all participants prior to the interviews. All information on emotions and experiences that came forward in the interviews was treated with respect and understanding. The interviewer made sure that all participants had peer support and/or service contact to handle any possible needs to process such emotions and experiences. No generative Artificial Intelligence programs were used in writing up this report.

## Results

The participant sample for the study presented in this report consisted of twelve women and twelve men between 25 and 62 years old. Four of them were under the age of 40 at the time of the interview, 17 were between 40 and 54 years old, and three were over the age of 55 (Table 1).

**Table 1:** Sample characteristics (numbers)

Gender:	
Female	12
Male	12
Age:	
20–29	1
30–39	3
40–49	12
50–59	7
60–69	1
Health region:	
South - East	7
West	12
Central	3
North	2
Years in LAR:	
1 – 4	4
5 – 9	6
10–14	3
15–19	8
20–24	3
Substitution medication at start of tapering:	
Methadone	9
Buprenorphine	14
Other	1
Status at the time of the interview:	
Left LAR – drug free	11
Left LAR – using drugs	1
Not started tapering	3
In the middle of tapering	3
Stopped tapering at a very low dose	6

Most of the 24 participants in the study had more than one episode of OMT treatment. The total number of years in LAR ranged from one to 20 years. Ten of the participants had been in the OMT program for more than 15 years, while only four had been in the OMT program for less than five years. At the time of the interview, 11 of the 24 participants had been out of the OMT program and had stayed stably drug-free for six months to five years, based on their self-reports. One participant had left the program but still used illegal drugs (mostly small amounts of buprenorphine). Six participants had re-entered or were still in the program but had ended up on a very small substitution medication dosage. Three participants were in the middle of a tapering process while three participants had concrete plans but had not yet started the tapering.

Fourteen of the participants were on buprenorphine as their last substitution medication when they started the tapering process or at the time of the interview (not started, temporarily stopped the tapering, or back in the program). Nine were on methadone and one was on buprenorphine depot. As this study was approved as a health service research project, Norwegian research regulations did not allow us to ask for other health and life situation information. In the presentation of results, we therefore primarily focus on how the participants experienced the process of being in the OMT program, leaving the program, or deciding that they had to remain in the program despite wanting to leave, and not on their health and life situation as such.

The 24 participants had very different experiences of all phases of the process of leaving OMT, which was to some degree related to the OMT program they came from. Experiences, however, also varied among participants from the same OMT program, at least in part as a function of relationships with individual program counsellors and their current life situation. These results are presented under five headings, related to the different phases of the process of leaving OMT. In the citations we use the Norwegian abbreviation LAR, for the OMT program.

### Longing for freedom – a new phase in life

The participants had mixed feelings about becoming a patient in the OMT program. On the one hand they saw how the OMT program had helped them to escape many of the negative consequences of active opioid addiction. Many also had positive experiences and benefited from being in the program:

*While I was in LAR*,* I qualified for higher education*,* and I had the care for children when they were small. So*,* I got to do a lot of things while I was in LAR.*

On the other hand, the OMT program, with its regulated substitution treatment and control systems, had given many of the participants a feeling of still being dependent, unfree and stigmatized:

*I started to realize that my stress-related problems were related to how I had to get my medication and take the urine tests*,* how I had to meet people I didn`t like and a lot of discomforting situations.*

Many participants reported a “longing for freedom”, from both the drug using life and from the LAR system:

*Freedom! Free to be more than a LAR patient. To be free to travel. To visit my son in another part of Norway*,* even on short notice. Not to have to go to the pharmacy every day*,* even on Sundays. I’m completely fed up - I’m so fed up with taking medicine.*

For most of the participants, this longing for freedom was also related to a feeling of having reached a new phase in their life. They felt ready to take a next step, and that step was to taper off their substitution medication and leave the program:

*I have felt healthy*,* so to speak. I’ve been at work*,* I’m a mother*,* so I sort of have a normal life. I got in touch with my family*,* and calmed myself down to build up a normal life again. Simply. I felt that then everything was somehow in place. It had done its job.*

When the participants told their counsellors in the OMT program that they wanted to leave, they got very different reactions. Many participants said that they observed systematic differences between different local programs or different regions, where the local program in one region was seen as fundamentally positive to the possibility of leaving, while attitudes in the other programs and regions differed from counsellor to counsellor.

Some of the participants who got very negative reactions to their expressed interest in leaving saw this as a sign of the traditional Norwegian OMT program view that it is not possible to leave OMT and remain stably drugfree. However, others more saw it as a sign of uncertainty among the counsellors:

*I think everyone was a bit afraid of it. Because every time I brought it up*,* it was a bit like that - yes*,* it will probably work*,* but are you sure that you want this. And*,* I understand that they ask those questions.*

#### The tapering process

The participants told differing stories about the tapering of their substitution medication, from very rapid tapers of only a few weeks to slow tapers over many years. One participant told a story about an extremely long taper:

*From the day I entered LAR*,* I managed perfectly well. But*,* for the first few years I was on very large – far too large – doses of methadone. And then I gradually tapered off in the last 15 years. But I could probably have gone out 7–8 years before I did.*

Participants also reported that a few tapers followed a fixed plan while most did not:

*I decided to quit and spoke to the doctor at LAR. He gave me a tapering plan. I don’t quite remember what the plan was*,* but it didn’t work. It was all too quick. So*,* I chose to spend longer time on that plan. So it lasted*,* I think the tapering lasted - instead of 6 weeks*,* it lasted for… 6 months.*

The potential complexity of the process of tapering was evident in the experience of one of the participants:

*I went on 85 mg Methadone. And*,* went down to 25 within a month and a half. Without any particular withdrawals*,* really. But*,* then the fear got the better of me and I continued on 25 mg for 8–9 months*,* maybe. And*,* at the very end*,* I had 2.5 in half − 2.5 in the morning and 2.5 in the evening. But then I delivered the rest of the medicine I had left and said now that’s enough. Now I can’t be bothered anymore*,* and it was July 24*,* 2018.*

Although the tapering process was complex and varied across individuals, there were two elements that were present in most of the stories. First, patients reported that they had to stop one or more times during the process, when they started to experience severe withdrawal symptoms. Second, the tapering of the last, often very small dose, often entailed strong withdrawal symptoms and fear of relapse to illegal drug use:

*I knew it was going to be difficult but not that the addict in me would grow that much again. That the troll would come up like that again as soon as I got withdrawal symptoms. It still came as a shock*,* even though I knew it rationally*,* right? If someone had come here and put heroin or something on the table*,* I would have taken it straight away.*

This fear of relapse that patients experienced during the tapering process was also felt by their families, both partners and children:

*So they were able to understand it*,* but at the same time they also couldn’t understand why I chose to go off those medications when they had actually gotten me drug-free. So*,* they (the children) were a bit worried.*

During the tapering process and often for a very long time after leaving treatment, the participants experienced severe withdrawal symptoms, sleeping problems, and both somatic and mental health issues. Some patients had long lasting problems which they experienced as withdrawal symptoms or as health problems caused by many years of substitution treatment:

*It has lasted for two years now*,* and for those two years I have had … abstinence and my body has spent a long time getting used to not being constantly tense*,* tingling in the feet and so on. Feelings that come back again*,* that ugly feeling.*

For others, the withdrawal symptoms rather quickly faded away, only to be followed by other underlying psychological problems:

*When I got off LAR*,* I thought*,* yes*,* the withdrawals are over*,* right? But*,* it’s not that*,* it was more the psychological bit. I felt when I was on methadone it was so much suppressed. It becomes so flat*,* in a way. And then when I left*,* a lot of emotions and sadness and longing and all this came up. So*,* it was much heavier than I had expected.*

Physical health problems were also common:

*Some purely physical things like that - increasing restlessness in the body*,* and then I had a very bad stomach for a couple of years - I still have that to a certain extent. I think that comes from having been on it* (the substitution medication) *for so long - of course.*

Participants indicated that these long-lasting psychological and physical health problems were the primary reasons they returned to the OMT program:

*Feeling the nerves that I get from not sleeping*,* I have coped very badly with that and it has probably meant that I will not get out of LAR. I’m not strong enough.*

### Coping and support

The participants’ experiences while tapering off the substitution medication show how big the challenges were that they had to face. To successfully transition off the program, the participants had to develop effective coping skills, and receive appropriate medical treatment and social support from others. The participants reported a range of strategies to cope with the short term and long-lasting problems they experienced:

*So*,* that’s what I did. Ate*,* went to meetings (NA)*,* showered*,* and went to meetings again (laughs).*

A few of the participants elected to go through the tapering without any withdrawal treatment medications to make it more bearable. However, most of the participants felt that an effective withdrawal treatment as well as proper medication for long lasting health problems were needed to achieve a successful transition off OMT:

*If I hadn’t been given some special medicine to take away the tingling*,* I almost don’t know if I would have lasted that long. And yes*,* I also got benzodiazepines.*

Some participants were also interested in taking alternative medications such as pregabalin or biperiden, and non-traditional drugs such as ibogain.

The participants had very different experiences regarding the help and support they got from the OMT program and other health and social services. Some reported a continuing and long lasting positive experience with all relevant services:

*I have received such good help from* (a treatment centre outside the OMT program). *I have received the help I need there and from the other health and social services. I have been listened to on the things I needed help with and received the help I needed.*

Other participants reported that they got more help from friends than the professional services:

*I did get some information through the* (municipality social service), *but I got most of it through friends. They are also drug-free. Information about work possibilities and about how to get back the driver license - all the information I’ve needed to get everything*,* I’ve been given from them.*

Some participants reported having a very difficult time as they left OMT :

*I was quite desperate. I didn’t have much follow-up from LAR. I was voluntarily discharged from LAR*,* finished. It was the General Practitioner (GP) I talked to a lot.*

The long contact with a General Practitioner (GP) was an important support for many of the participants:

*I have a good relationship to my GP. He has followed me through all these years. He knows me*,* and I have always been honest with him*,* open with him about everything. So*,* he knows my whole life situation and my problems.*

When participants were asked about how to improve support in the tapering process, three recurrent themes emerged. Some participants felt that the detoxification units they were admitted to were not the right places for them. The other patients in these units were in a process of detoxification from illicit drugs rather than substitution medication, and the relationships with these patients were often problematic. The health professionals in these units were skilled when it came to illicit drugs detoxification, but often lacked competence when it came to tapering from substitution medication.

Secondly, many participants felt that they did not receive the extra support needed during the last phase of the tapering. Some had experiences with peer networks that offered support and activities like yoga. Others had been to a private resort that offered a sheltered and supportive environment, as well as activities like yoga and hiking, and this setting was appealing to many in our sample:

*I was going to start with yoga and I’m thinking of starting in* (a peer organized yoga organization). *I would have travelled to* (the resort) *on the day if I had had the opportunity.*

The third kind of support that had been important for many of the participants were specialized treatment services to deal with all the mental problems that again came to the surface after tapering off the substitution treatment:

*It was when I was in (mental health) treatment that I started to get the pieces together and having a normal life.*

Others were met with quite another attitude:

*If I applied for (substance use or mental health) treatment*,* in-patient treatment*,* I were only told that*,* no*,* you have received treatment - in LAR.*

### Continue the substitution treatment?

As earlier mentioned, most of the participants had been in and out of OMT program several times before. Typically, they had been offered treatment again after being discharged or discharging themselves and then relapsing to short or longer periods of heavy drug use. All of the participants emphasised the importance of being able to temporarily stop their taper or increase the dose before continuing the taper, or to stabilize the treatment on a much lower dose rather than completing the taper:

*After a long*,* long time tapering off Subutex*,* I switched to Temgesic*,* and then switched to Norspan. So I did everything by the book*,* took a long time*,* but still I completely panicked about taking the step to zero. And it ended up that I rather started going up a bit again.*

For six of the participants, the current attempt to taper the substitution treatment ended in re-entering or continuing in the OMT program, but on a much lower dose and without any control measures:

*LAR as a system*,* it does not characterize my life today at all. I have nothing to do with them. They sort of prescribe my meds*,* and I pick up for a month at a time.*

For these participants, the ultimate aim could still be to leave the OMT program, but with the safeguard of remaining on a small dose of substitution medication, without the feeling of being locked into and dependent on a controlling system.

*I’m on a small dose of subutex*,* and I’m only doing that because I haven’t been able to come off. It’s not that I need to not use opioids. But I won’t know that until I leave LAR. But I think very little about drugs and I work and actually have a very good life.*

But even if they felt that they now had a good life in the OMT program, some still had a bad feeling of still being addicted to opioids:

*I want to speak very highly of LAR. Which has given me a lot of opportunities. And the trust. And today there are no restrictions on being in LAR. Other than the fact that I know that I am addicted to opioids. And I want to live a life without pushing myself strong drugs every day. Right?*

### Life after leaving

When talking about leaving the OMT program, most participants were talking about tapering off the substitution treatment and living a stable drug-free life. But for some of the participants, leaving the OMT program also meant losing other services delivered by the program or related to or organized by other organizations. For some this meant the support they had been given by people in the program:

*But I do miss that (support) a bit. I’ve had the people there with me for 20 years. And suddenly like that… so I’ve actually been allowed to continue the contact with my LAR counsellor for an extra year*,* even if I`m out of the program.*

For others, the reduced access to free dentist services was particularly problematic:

*The social service agreed to cover something. But it was only the very “basic” so that I don’t look like… an ex-addict. So you get a free dentist when you’re in LAR*,* but if you’ve left LAR*,* you’ve still got the same consequences from many years of drug use.*

However, these problems were not large or significant enough to overshadow the generally very positive experience of having left the program and begun to live a drug free life. Most of the participants that have reached this goal talked about the importance of paid work, of engagement in peer work and in meaningful leisure activities, and about their new social life:

*I have been lucky. Because I have good contact with my family. Visit them regularly. I have a good network*,* I have good friends that I have had since I was young*,* in a way. They have always been there.*

Many participants talked about plans for the future:

*And*,* then I go to school. I’m studying - I’ve finished my first year of education to be a health worker and now I’m studying my second year.*

This quote sums up how many of the participants felt about leaving OMT and living a stable drug free life:

*It is absolutely magical. I feel like – yes*,* a great liberation.*

## Discussion

Maintenance treatment saves lives and makes it possible to start a process of health and social rehabilitation. However, for many OMT patients, treatment with substitution medication has become the sole aim of their life, and being in OMT gives many patients the desperate sense that they have reached a last resort in life [20]. The drug administration routines and control measures thought necessary to secure a medically sound treatment and stop leakage to the illegal drug market lead many patients to feel that they have exchanged a life with dependence on illegal opioids for a stigmatized and controlled life as an OMT patient [4, 16, 28]. This is the paradox of OMT, which leads many patients to want to leave OMT programs [1, 19, 31, 38].

Most patients who leave OMT, for any reason, relapse to new opioid use after a short time, with a high risk of overdose and other health and social problems [8, 34, 37]. Developing more effective means of maintaining higher rates of retention in treatment has become an important research topic, and clinical guidelines have traditionally included strong and general advice against leaving substitution treatment [23]. Most research and clinical practice characterize severe opioid addiction as a chronic disease and lifelong substitution maintenance treatment as the preferred treatment. When health and social rehabilitation is introduced as a goal, it is often based on the assumption that addiction has to be understood as a dynamic, and often long lasting bio-psycho-social disorder [22], which can be sent into remission by behaviour-change and changes in health and social life situations. So, it is problematic to conclude that all rehabilitated OMT patients need continued substitution medication, the core reason to continue as a OMT program patient. This is also the reason why the national clinical guidelines for the Norwegian OMT program in 2022 were changed from a strong and general advice against leaving the program to a more qualified formulation: “Patients who after assessment together with a doctor want to taper off their substitution medication, should be offered gradual and long-term tapering, with adapted psychosocial follow-up and rapid dose increase if necessary.” ([17], p. 72). The basis of this new guideline is the research showing that a small but significant share of those that leave OMT can stay stably drug free, but also that this, to a large degree, is dependent on having years of a drug free life while on substitution treatment and having a good psychosocial life situation.

The participants in this study who wanted to leave the Norwegian OMT program felt that they had reached a new phase in their lives, where they could manage without the substitution medication and the restrictions of the OMT program. We have investigated the experiences of these patients, as they asked for support to taper off the medication and cope with the health and social challenges they encountered in the process of trying to leave the OMT program. In relation to previous studies [32, 40, 43], this study adds new knowledge related to two themes: (1) how to deal with the challenges related to the tapering of the substitution medication, and (2) what is required to achieve the goal of leaving the OMT program and live stable drug free lives.

### The tapering process

Most research on planned processes of leaving OMT focuses on the tapering off the substitution medication. Some studies have had the aim of developing tapering decision algorithms or guidelines [30, 43], while others have made more general conclusions, finding that gradual dose reductions interspersed with periods of stabilization increase the possibility of a successful tapering [27]. The participants in this study focused more on the need for a close dialog between the patients and the OMT program service rather than following a strict algorithm, through the whole tapering process. No two processes seemed to be alike. The length of the process varied from weeks to years. Only a very few individuals could successfully adhere to a fixed tapering plan. Conversely, most of the participants needed slowing down at some points, stopping the tapering, and even increasing the dosage before again continuing the tapering process. So, instead of trying to develop and present fixed tapering plans for the patients to follow, patients benefited more from adaptive routines that relied on dialog between them and their service providers throughout the process, which took into account individualized goals and patient experiences each step on the way.

Based on the experiences of the participants in this study, it is possible to identify several issues where service provider competence seemed to be lacking and where current services seemed to be inadequate. Many participants thought that the ordinary detox service units, which were primarily filled with patients trying to stop using illegal drugs, were not a constructive treatment environment for them. They also believed that the staff members in these units were lacking competence in treating patients that were in a process of tapering off substitution medications. They felt that the withdrawal symptoms from tapering off substitution medication were more severe and different from those they experienced when they stopped using illegal drugs. Some participants also had suggestions for alternative medication to what they were offered. Many participants felt that the services seemed to lack both interest and competence regarding what were the most effective medications to use in detoxification from substitution medication. Some participants also had the same experience when it came to medication for long term withdrawal problems, long lasting health problems related to both the use of illegal drugs and substitution medication, and when it came to underlying health problems surfacing after tapering off the substitution treatment. Recent research on opioid withdrawal treatment could have added valuable knowledge to the services on how to deal with these problems [12].

Medical treatment, however, was not the only important intervention needed in dealing with the challenges facing the patients in the tapering process. The last phase of the process, transitioning down to a very low dose, was quite hard and getting all the way down to zero was extremely difficult. In this phase, short-term withdrawal symptoms reached their peak and all other somatic and mental health problems came to the surface. In this situation, many participants felt in need of some kind of sheltered environment or retreat that could offer support from people who had been or were in the same situation, traditional medical and psychosocial treatment, and other, less traditional interventions, such as yoga, physical activities, art- and animal-assisted therapies.

One of the strengths of the Norwegian OMT program is how it in, most cases, offers long lasting and continuing relationships with an interdisciplinary team of counsellors, and how both other specialized out-patient services, municipality services and GPs are organized to offer the same kind of continuing health and social services. In most cases when the contact with these services is lacking or not experienced as supportive, then this is most of often due to problems in the personal relationship between patient and caregiver or bad collaboration between different services. Some participants had bad experiences with counsellors in some of the OMT program centres, which were not interested in using resources for inpatient treatment of severe mental problems surfacing during and after the tapering. Some treatment rights, such as rights to free dental treatment are only offered when one is an OMT patient or being transferred to other specialized services. This seems to be a yet another paradox of being a patient in the OMT program; becoming a patient in the program makes it possible to get access to services to deal with other health and social problems that are common in people with opioid use disorder. By tapering off the substitution treatment, and by that also leaving the OMT program, the participants in the study risked losing many of the other health and social services they still needed, as well. It is as if being in the role as an OMT program patient justifies the need for other treatments and services, and leaving the program often means that these other services are no longer available.

### Good outcomes

To become stably drug free after a severe opioid addiction and substitution treatment is extremely difficult and challenging, but not impossible. A sample of participants in a qualitative study like this does not provide information on what proportion of patients in maintenance treatment have the opportunity to leave the treatment or the proportion that become stably drug free. This study does, however, challenge both a one-sided view that severe opioid addiction is a chronic disease in the same way as insulin dependent diabetes, always in need of substitution treatment to stay drug-free, and the one-sided view that it is primarily due to social and psychological maladjustments, always making it possible to go off the substitution treatment as soon as health and social issues have been addressed. This study found that there were at least some patients who have had a long-term history of severe opioid addiction but who after reaching a high level of health and social rehabilitation were able to leave the substitution treatment and live a stable drug free life. No type 1 diabetic can quit the insulin treatment in the same way. This study also shows that other patients who seemed to have reached the same level of rehabilitation, found that their opioid dependence was so strong that they were unable to leave the treatment. It also shows that for some a very low substitution drug dose is a safeguard they still need to achieve their primary goal of having a free life and not being labelled by themselves and others, as an OMT program patient.

This study was approved as a health service research study, focusing on the participants own experiences of leaving the OMR program. This means major restrictions on what personal information was allowed to be collected. Therefore, it is not possible to answer the question whether those who had left the program and become stably drug-free were initially better prepared to leave the program than those who are still in the program. The study does, however, points out how differences in the experiences with the substitution drug tapering and with the support they received from health and social services and family and friends in the process, played a very important role in being able to leave the program and stay stably drug free.

The different outcomes seen in this study are also reflected in the new clinical guideline for the Norwegian OMT program, as stabilization on a lower maintenance dose or termination of the treatment are defined as equal goals of the tapering. Previous clinical guidelines and discussions have most often lined up two alternatives for the OMT program patients: Either continue a safe treatment on high substitution medication dosages or leave with the high risk of relapse and overdose. The experiences of the six participants who ended the tapering on a low substitution medication dosage show that the safeguard of a small substitution medication dose combined with a medication regime not different from most other drugs, can fulfil the aims of initially wanting to leave the OMT program.

### OMT at a turning point?

The Norwegian OMT program and OMT in many countries seem to be at a turning point. The Covid-19 pandemic made it impossible to continue many of the restrictive procedures for delivering the medication, and new routines were developed, more based on trust than on control [21, 36]. Increased demands on user involvement in treatment point in the same direction. The revision of the Norwegian national OMT guidelines in 2022 underline the need for more active user engagement in the treatment, such as choosing between substitution medications and basing the treatment program more on trust than on control. The last Norwegian OMT national conference showed that the OMT program departments and the health professionals seem to be on their way to implementing these parts of the new guidelines.

But when it comes to the new guideline on how the services should react to wishes to leave substitution treatment and support the patients during and after the leaving process, the experiences of many of the participants in this study show that many of the services seem not to have taken much notice. On the other hand, the experiences also show that some services and some health professionals have already developed attitudes and practices in line with the new guideline. The experiences of the participants in this study show what it takes to leave the OMT program and live a good and stable drug-free life. They also show that it is possible to achieve the aims of wanting to leave the program by ending up with the safeguard of a low substitution medication dose. In this way, this study supports both the recommended aims and measures of the new Norwegian national OMT guideline.

## Strengths and limitations

The main strength of this study is the co-research process that produced the richness of the qualitative data on which it is built. Twenty-four participants who all had wanted to leave the OMT program shared their experiences on all aspects of being in the program, wanting to leave the program, and having left or remained in the program. The experiences are both rich and varied. Some participants had not yet started the process of trying to leave, some were in the middle of the tapering process, some had chosen to stay in the program but on changed terms, and almost half had gone through the whole tapering process and had lived stable drug free lives for periods ranging from several months to several years. It is also a strength that the participants were recruited in all the major health regions of Norway, meaning that the participants had experiences of being in or having left the role as patient in many different OMT program departments. And it is a strength that none of the OMT program patients / former program patients that were asked to participate, declined to participate.

The recruitment process is, however, also the weakness of this study. In one region, the OMT program department in one hospital was able to mediate contact with many potential participants, which led us to other potential participants. OMT program departments in the other regions were also asked to help in the recruitment, but none was able to do so. This meant that contacts in user organizations in these regions had to help us contact potential participants. Some of them again helped us contact other participants. The difference in the ability of the OMT program departments to recruit participants to the study could be interpreted as a difference in how the different departments think about the issue of leaving the OMT program, but it could also be a result of differences in the relationship between the researchers and different OMT program departments. It is difficult to determine whether these differences have led to a systematic bias in the participants sample of the study. However, it is quite possible that our recruitment methods were more likely to enroll patients who had more favourable outcomes in their attempts to come off of maintenance opioid medications than average patients in these circumstances.

## Conclusions

To become stably drug free after a severe opioid addiction and substitution treatment is extremely difficult and challenging, but not impossible. This study shows that patients with a strong motive to free themselves from the extraordinary treatment regime of the Norwegian OMT program, and who have reached a new life phase with better control over their health problems and a socioeconomic stability, are able to leave the program and become stably drug free. It also shows that some individuals in a similar life situation still experience their opioid addiction to be too strong to entirely quit the substitution treatment. Many of them can, however, stabilize on a low substitution medication dosage and live a life not dominated by extraordinary medication and control regimes. The study shows that LAR patients who want to leave the program are met with varied attitudes and levels of help and support within different programs. It also uncovers a large need for building up better tapering support, improved and more individualized services to help with the tapering of the substitution medication and the health and social problems facing patients in the process. After a long period of a drug-free life and a good level of health and social rehabilitation, and given good treatment and support, many OMT program patients are in a good position to leave the OMT program or continue the substitution treatment in a less OMT program-dominated life.

## Supplementary Information

Supplementary Material 1.

## Data Availability

The audio files and transcripts of all interviews are available on the research centre’s server.
