# Supplementary material for: Leaving the Norwegian opioid maintenance treatment program - patient experiences
Source: BMC Health Serv Res. 2024 Nov 25;24:1464. doi: 10.1186/s12913-024-11859-3 (PMC11590507; doi:10.1186/s12913-024-11859-3)
Supplement: Supplementary file 1 — Supplementary Material 1. [file 12913_2024_11859_MOESM1_ESM.docx]

**Leaving N-OMT: Interview guide**

**Alternative adapted to people who want to leave N-OMT, but who have not yet left:**

• Male/female

• How old are you?

• Where are you in the process

• How many years have you had a serious drug problem - in total and with the use of opiates?

• How long have you been in N-OMT (Total and now the last time, if several periods)?

• Have there been any changes in your N-OMT treatment since you now want to leave N-OMT?

• Why do you want to leave N-OMT - conditions that have made it difficult to continue in N-OMT?

a. Changes in your health situation

b. Changes in your life situation: changes in housing situation, work, network, contact with family, hobbies / interests, structure in everyday life etc. ?

c. The experience of the treatment in N-OMT (medication, check-up routines, pick-up arrangement)

d. Would you continue in N-OMT if something in the N-OMT treatment had been different?

• What type of problem do you envisage when you leave N-OMT - in the short and long term?

a. Abstinence, addiction?

b. Other health problems?

c. Problems related to your life situation in general?

• Who do you imagine should help you deal with/solve these problems?

• Can you cope with these problems from where you currently live or do you think you should live/stay somewhere else, get help somewhere else?

• How do you imagine a life without N-OMT?

a. Your health situation – need for further treatment?

b. Your life situation: changes in housing situation, work, network, contact with family, hobbies / interests, structure in everyday life etc. ?

**Alternative adapted to people who have experience of leaving N-OMT**

• Male/female

• How old are you?

• Where do you stand in the process

• How many years have you had a serious drug problem - total and with the use of opiates?

• How long have you been in N-OMT (Total and now the last time, if several periods)?

• Were there any changes in your N-OMT treatment in the period before you left N-OMT?

• Why did you want to leave N-OMT – conditions that made it difficult to continue in N-OMT?

a. Changes in your health or life situation?

b. The experience of the treatment in N-OMT (medication, control routines, pick-up arrangement)?

c. Would you continue in N-OMT if something in the N-OMT treatment had been different?

• What type of problem did you experience when you left N-OMT - in the short and long term?

a. Abstinence, addiction?

b. Other health problems?

c. Problems related to your life situation in general?

• Who have you received help from to deal with/solve these problems?

• Did you deal with these problems where you lived or did you have to live/stay somewhere else, get help somewhere else?

• What did you need help for/who did you need help from?

• How do you imagine a further life without N-OMT?

a. Your health situation – need for further treatment?

b. Your life situation: changes in housing situation, work, network, contact with family, hobbies / interests, structure in everyday life etc. ?

• If you are now back in N-OMT and/or in a life with serious drug problems: What made you unable to get out of N-OMT / ended up back in a life with serious drug problems?
